# Inferior mesenteric artery ligation level in rectal cancer surgery: still no answer—a systematic review and meta-analysis

**DOI:** 10.1007/s00423-023-03022-z

**Published:** 2023-07-26

**Authors:** Roberto Cirocchi, Francesco Marchetti, Giulio Mari, Francesco Bagolini, Davide Cavaliere, Stefano Avenia, Gabriele Anania, Giovanni Tebala, Annibale Donini, Richard Justin Davies, Abe Fingerhut

**Affiliations:** 1 Department of Medicine and Surgery, University of Perugia, Perugia, Italy; 2 Department of Surgical Sciences, University of Ferrara, Ferrara, Italy; 3 Department of Colorectal Surgery ASST Brianza, Desio Hospital, Desio, Italy; 4 Department of Colorectal Surgery and General Surgery, Ospedale Per Gli Infermi, Faenza, Italy; 5 Department of Digestive and Emergency Surgery, Hospital of Santa Maria of Terni, Terni, Italy; 6 Cambridge Colorectal Unit, Addenbrooke’s Hospital, Cambridge University Hospitals NHS Foundation Trust, Cambridge, UK; 7 Department of General Surgery, Ruijin Hospital, Shanghai Jiao Tong University School of Medicine, Shanghai Minimally Invasive Surgery Center, Shanghai, China; 8 Section for Surgical Research, Department of Surgery, Medical University of Graz, Graz, Austria

**Keywords:** Rectum, Laparoscopy, Surgery, Rectal cancer, IMA ligation

## Abstract

**Objective:**

The aim of this systematic review and meta-analysis is to summarize the current scientific evidence regarding the impact of the level of inferior mesenteric artery (IMA) ligation on post-operative and oncological outcomes in rectal cancer surgery.

**Methods:**

We conducted a systematic review of the literature up to 06 September 2022. Included were RCTs that compared patients who underwent high (HL) vs. anterior (LL) IMA ligation for resection of rectal cancer. The literature search was performed on Medline/PubMed, Scopus, and the Web of Science without any language restrictions. The primary endpoint was overall anastomotic leakage (AL). Secondary endpoints were oncological outcomes, intraoperative complications, urogenital functional outcomes, and length of hospital stay.

**Results:**

Eleven RCTs (1331 patients) were included. The overall rate of AL was lower in the LL group, but the difference was not statistically significant (RR 1.43, 95% CI 0.95 to 2.96). The overall number of harvested lymph nodes was higher in the LL group, but the difference was not statistically significant (MD 0.93, 95% CI − 2.21 to 0.34). The number of lymph nodes harvested was assessed in 256 patients, and all had a laparoscopic procedure. The number of lymph nodes was higher when LL was associated with lymphadenectomy of the vascular root than when IMA was ligated at its origin, but there the difference was not statistically significant (MD − 0.37, 95% CI − 1.00 to 0.26). Overall survival at 5 years was slightly better in the LL group, but the difference was not statistically significant (RR 0.98, 95% CI 0.93 to 1.05). Disease-free survival at 5 years was higher in the LL group, but the difference was not statistically significant (RR 0.97, 95% CI 0.89 to 1.04).

**Conclusions:**

There is no evidence to support HL or LL according to results in terms of AL or oncologic outcome. Moreover, there is not enough evidence to determine the impact of the level of IMA ligation on functional outcomes. The level of IMA ligation should be chosen case by case based on expected functional and oncological outcomes.

**Supplementary Information:**

The online version contains supplementary material available at 10.1007/s00423-023-03022-z.

## Introduction

Rectal cancer remains one of the most common gastrointestinal malignancies, and its prevalence is likely to increase in the near future [1]. Many patients diagnosed with rectal cancer undergo low anterior resection (AR) or abdomino-perineal excision (APE) of the rectum [2]. There is widespread surgical consensus on the essential role of achieving an intact excision of the mesorectum with adequate, clear circumferential and longitudinal margins [3]. These criteria have a direct impact on overall survival (OS) and disease-free survival (DFS) [3]. The same level of consensus is yet to be reached regarding proximal or distal ligation of the inferior mesenteric artery (IMA) during these surgical procedures, mainly because of the technical and functional implications that this ligation entails. The optimal level of ligation of the IMA during reconstructive surgery for rectal cancer remains a topic of discussion and debate.

During rectal resection, the IMA can be ligated proximally (high ligation) or distally to the left colic artery (low ligation), but the impact of the level of ligation of the IMA in terms of post-operative complications and oncological results is still an important dilemma and has given rise to several protocols [4–9], meta-analyses, and prompted specific recommendations by international societies [10–12]. Also, our previous review [9] did not allow definitive conclusions on the optimal level of IMA ligation in colorectal cancer surgery due to the poor quality and high heterogeneity among the comparative studies available in the literature at that time. No significant difference has been found between high and low ligation in regard to surgical complications and short-term oncological outcomes [13–16], but data regarding functional outcomes, in particular the genitourinary system, and long-term oncological results are still lacking.

The debate between high and low ligatures of IMA is not new; however, whereas earlier systematic reviews and meta-analyses were primarily based on observational comparative studies, recently published reports have only included a small number of RCTs and have only analyzed oncological or functional outcomes. Even though during the last 5 years many systematic reviews and meta-analyses were published on this topic, recently the results of numerous RCTs were made available in the literature, so we compiled the most recent scientific evidence regarding the influence of the level of IMA ligation on post-operative and oncological outcomes in rectal cancer surgery.

## Materials and methods

The study protocol for this systematic review and meta-analysis (CRD42021241774) was registered with the PROSPERO database (http://www.crd.york.ac.uk/prospero).

**Fig. 1:**
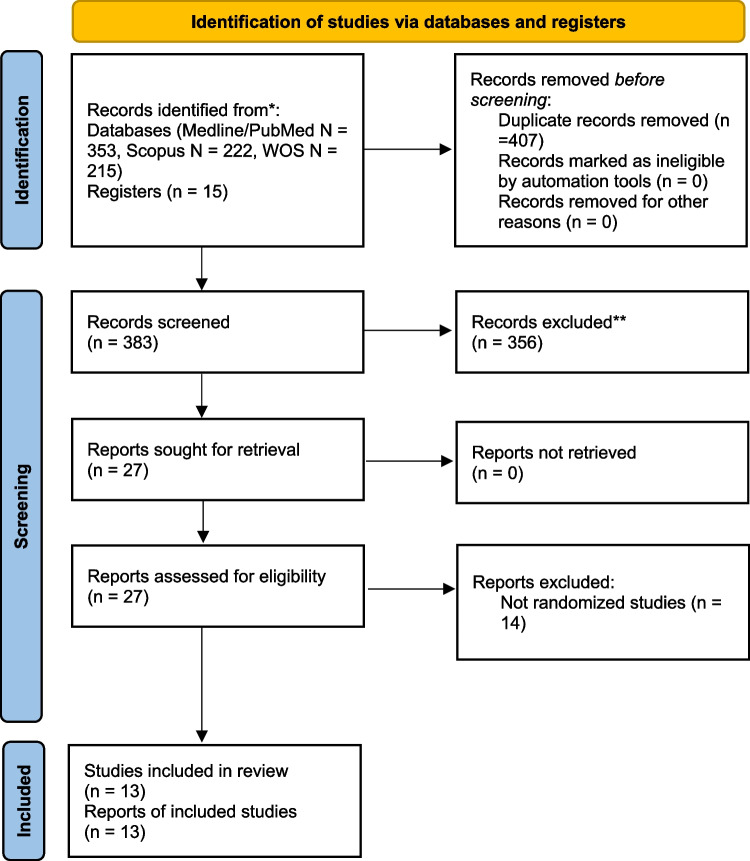
PRISMA flow diagram of systematic review. *Consider, if feasible to do so, reporting the number of records identified from each database or register searched (rather than the total number across all databases/registers). **If automation tools were used, indicate how many records were excluded by a human and how many were excluded by automation tools

We conducted a systematic review of the literature up to 06 September 2022 according to the Preferred Reporting Items for Systematic Reviews and Meta-Analyses (PRISMA) guidelines [17] (Fig. 1).

All studies included in this systematic review were randomized clinical trials (RCTs) that compared patients who underwent high vs. low IMA ligation during resection for rectal cancer. Patients treated with abdomino-perineal excision of the rectum were excluded; only patients undergoing AR were selected.

Our comprehensive search of the literature was performed by analyzing Medline/PubMed, Scopus, and the Web of Science (Web of Science Category: Surgery and Oncology) databases without any language restrictions.

The references of all included studies were hand-screened to identify any studies missed during the initial search.

In Medline/PubMed, the combinations of the following MeSH terms were used: “ligation” AND “high” OR “low” AND “inferior mesenteric artery” AND “surgery.”

The SCOPUS search strategy included ligation AND “inferior mesenteric artery” AND “high” OR “low” AND surgery.

The WoS search terms were “ligation” AND “inferior mesenteric artery.”

The “related articles” function from PubMed was used to broaden the search, and the reference lists of all eligible studies were reviewed. To minimize the retrieval bias, a manual search was performed through the Google Scholar database. A search for ongoing clinical trials was performed on ClinicalTrials.gov. Unpublished data was excluded.

Two authors (RC and FM) evaluated the titles and abstracts of all articles included in the search. Subsequently, the complete text of these studies was evaluated, and the following information was collected: year of publication, study design, inclusion criteria, exclusion criteria, and outcomes. The primary endpoint was overall anastomotic leakage (AL) analysis.

Secondary endpoints were an overall number of harvested lymph nodes, number of vascular root lymph nodes, 5-year overall survival (OS) (rate) and 5-year disease-free survival (DFS) (rate) laparoscopic-to-open conversion rate, operative time (minutes), estimated operative blood loss (ml), urinary retention rate, sexual dysfunction rate (on the basis of the International Index of Erectile Function (IIEF) score 9 months after rectal resection), and duration of hospital stay (days).

Dichotomous variables were expressed by risk ratios (RR, with the HL as the “exposure” criterium in the RR calculation), while continuous variables were expressed by weighted mean differences (WMD). Meta-analyses were performed with the randomized Mantel–Haenszel method (random effect). All results were reported as a forest plot. Heterogeneity was analyzed with the *I*^2^ statistic and Cochrane’s. Data analysis was performed using Review Manager meta-analysis software (RevMan) v. 5.4.1 (Copenhagen: The Nordic Cochrane Centre, The Cochrane Collaboration, 2018).

The risk of bias in the included studies was evaluated by two authors (FM and FB) according to the RoB 2 revised tool for assessing the risk of bias in RCTs [18].

## Results

As indicated in the PRISMA flowchart (Fig. 1), the initial search produced 790 potentially relevant articles. After screening the titles and abstracts, we evaluated the full text of 27 articles: 14 articles were not randomized studies [19–32], consequently they were eliminated, and in the remaining 13 articles [33–45], four papers reported the results for the same two RCTs, in particular the HTLT trial [35, 36] and the HIGHLOW trial [37, 40]. Therefore, eleven RCTs were included in this systematic review and meta-analysis.

### Study characteristics

The study enrollment period ranged from 2006 [35] to 2018 [33]; the sample size ranged between 46 [34] and 331 [35] patients. Eight studies were performed in Asia (1006 patients, 73%), including seven from China (675 patients) and one from Japan (331 patients). Three studies were performed in Europe (372 patients, 27%), including two conducted in Italy (242 patients) and one in Poland (130 patients). The pooled data included 1331 patients (range per study: 10–1100 patients) who were planned to receive either high IMA ligation (HL, 666 patients) or low IMA ligation (LL, 665 patients). A total of 1143/1331 (85.8%) patients underwent laparoscopic rectal resection (1143 patients), while 88 had an open rectal resection. Data on the modality of surgical access was not available for 100 patients (Table 1).

**Table 1:** Included randomized controlled trials

Author and year of publication	Nation	Type of study	No. of patients included	Time of enrolment	Type of access	Location of cancer
Feng et al. (2021)	China	RCT	95	2016–2018	LA	Rectum
Kruszewski et al. (2021)	Poland	RCT	130	2010–2016	OA	Rectum
Fiori et al. (2020)	Italy	RCT	46	2013–2019	LA	Rectum
Mari et al. (2020–2022)	Italy	RCT	196	2014–2016	LA	Rectum
Fujii et al. (2019)	Japan	RCT	331	2006–2012	OA-LA	Rectum
Guo et al. (2019)	China	RCT	57	2013	LA	Rectum
Matsuda et al. (2017)	Japan	RCT	100	2008–2011	OA-LA	Rectum
Niu et al. (2016)	China	RCT	97	2009–2015	LA	Rectum
Wang et al. (2015)	China	RCT	128	2012–2013	LA	Rectum
Wu (2017)	China	RCT	96	2014–2016	LA	Rectum
Zhuo et al. (2018)	China	RCT	102	2015–2016	LA	Rectum

*RCT*, randomized control study

Type of approach: LA, laparoscopic assisted; *OA*, open access

Pooled trials showed no statistically significant differences for age, gender, BMI (body mass index), ASA (American Society of Anesthesiologists) score, and TNM stage. Among the examined studies, patients with stage IV disease were excluded, except for four analyses [35, 36, 40, 41]; the other studies included only patients with stage I, II, and III disease (Table 2).

**Table 2:** Characteristics of patients in included studies

Author and year of publication		Patients enrolled	Age mean (SD)	Sex (M/F)	BMI mean (SD)	ASA (I, II, III, IV)	T stage (I, II, III, IV)
Feng 2021	High ligation	47	60.5 ± 10.2	26/21	22.6 ± 2.3	NR	TNM: 21, 12, 14, 0
	Low ligation	48	59.8 ± 8.9	24/24	22.9 ± 2.7	NR	TNM: 25, 13, 10, 0
Kruszewski et al. (2021)	High ligation	65	64 ± 9	37/28	27 ± 4	NR	TNM (0, I, II, III, IV): 10, 22, 14, 19, 0
	Low ligation	65	65 ± 8.5	34/31	27 ± 4	NR	TNM (0, I, II, III, IV): 7, 16, 18, 24, 0
Fiori et al. (2020)	High ligation	22	68 ± 9	12/10	NR	0, 18, 4, 0	TNM: 22, 0, 0, 0
	Low ligation	24	68 ± 11	14/10	NR	1, 16, 7, 0	TNM: 24, 0, 0, 0
Mari et al. (2020)	High ligation	111	67 ± NR	65/46	26.7 ± 4.6	NR	TNM: 44, 25, 39, 3
	Low ligation	103	68 ± NR	63/40	26.1 ± 3.9	NR	TNM: 60, 21, 19, 3
Fujii et al. (2019)	High ligation	107	66 ± NR	68/39	23.1 ± NR	29, 74, 4, 0	TNM (0, I, II, III, IV): 3, 45, 20, 36, 3
	Low ligation	108	66 ± NR	68/40	22.3 ± NR	53, 95, 12, 0	TNM (0, I, II, III, IV): 5, 43, 20, 36, 4
Guo et al. (2019)	High ligation	29	NR	NR	NR	NR	NR
	Low ligation	28	NR	NR	NR	NR	NR
Matsuda et al. (2017)	High ligation	51	69 ± NR	33/18	NR	NR	TNM (0, I, II, III, IV): 2,7,15,23,4
	Low ligation	49	67 ± NR	34/15	NR	NR	TNM (0, I, II, III, IV): 0, 17, 17, 13, 2
Niu et al. (2016)	High ligation	45	49.9 ± 8.2	25/20	NR	NR	TNM: 14, 22, 9, 0
	Low ligation	52	51.3 ± 6.3	27/24	NR	NR	TNM: 19, 25, 8, 0
Wang et al. (2015)	High ligation	63	56.8 ± 14.2	NR	NR	NR	NR
	Low ligation	65	58.6 ± 13.7	NR	NR	NR	NR
Wu (2017)	High ligation	50	58.4 ± 9.3	NR	NR	NR	NR
	Low ligation	46	59.1 ± 9.1	NR	NR	NR	NR
Zhou et al. (2018)	High ligation	52	52.7 ± 12.9	NR	NR	NR	NR
	Low ligation	52	53.9 ± 14.5	NR	NR	NR	NR

Inclusion and exclusion criteria were reported and were referred to as having the same characteristics in the various studies.

### Quality assessment

The risk of bias for each trial is indicated in SDC2. However, the analysis does not report differences in the characteristics between the two groups, which might suggest the absence of bias associated with the randomization process. Blinding of evaluators was reported in 3 RCTs [33, 40, 41], while patients were not aware of their assigned intervention [38] in only one study.

The overall risk of bias was deemed to be “low” for 4 RCTs [33, 35, 37, 40] (two of which were referred to the same study), showed “some concerns” for 3 RCTs [34, 40, 41] and was “high” for 1 [38], according to the RoB 2 revised tool for assessing the risk of bias [18].

### Primary outcome

#### Anastomotic leakage (AL)

**Fig. 2:**
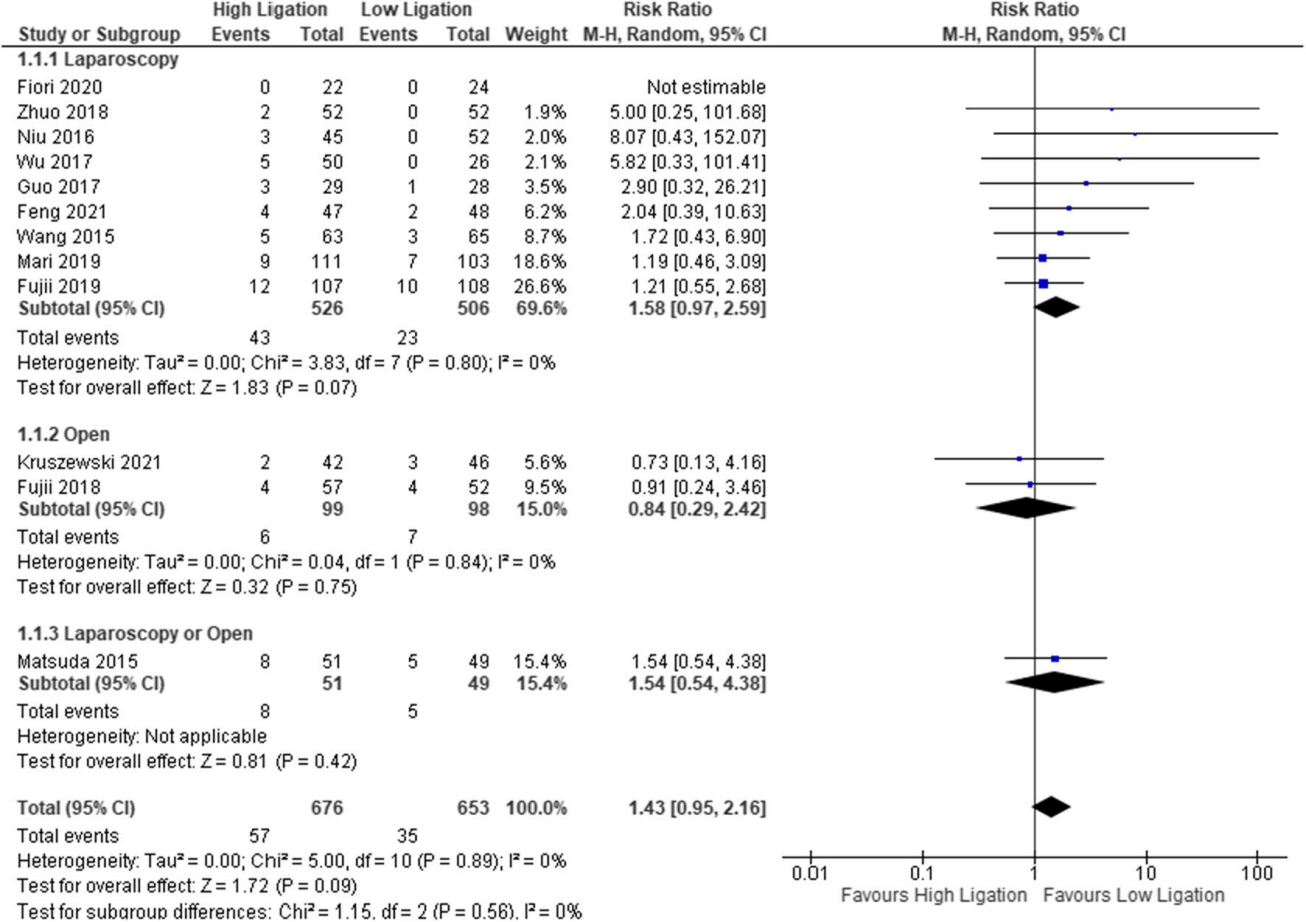
Anastomotic leakage (AL)

All 11 RCTs reported this outcome (participants = 1329). Although the overall prevalence of AL was lower in the LL group (5.36%, 35/653) when compared to the HL group (8.43%, 57/676), the difference was not statistically significant (RR 1.43, 95% CI 0.95 to 2.96). *I*^2^ presents the inconsistency between the study results and quantifies the proportion of observed dispersion due to between-study differences [46]. The RR of AL in the subgroup of the eight laparoscopic studies (1097 patients) favored LL (4.54%, 23/506, LL vs. 8.17%, 42/494 HL), but the result was not statistically significant: 1.58 (95% CI 0.97 to 2.59); on the other hand, the RR of AL for open surgery was lower in the HL group (6.06%, 6/99 for HL) vs. LL (7.14%, 7/98), but the result was not statistically significant: 0.84, 95% CI 0.29 to 2.42) (Fig. 2, SDC3).

The funnel plot shows an asymmetrical shape, indicating the presence of bias. In the laparoscopic group, the asymmetrical shape favors LL in the lower-weight RCTs rather than higher-weight RCTs.

In three studies, the authors did not report the type and grade of AL (277 patients: Niu et al. [42] 3/45 HL vs. 0/52 LL, Wu [44] 5/50 HL vs. 0/26, Zhou et al. [45] 2/52 HL vs. 0/52 LL); nine studies (1052 patients) reported grade B or C of AL. The analysis of grade B or C of AL (9 studies, 1059 patients) showed that the risk of AL in HL was higher than in LL ((8.89%, 47/529) vs. 6.69%, 35/523) (HL/LL), but the RR was not statistically significant (RR 1.31, 95% CI 0.86 to 2.00). Although the excluded studies included only laparoscopic rectal resections, the new subgroup analysis (6 studies, 755 patients) [33–35, 37, 38, 43] did not find any statistically significant difference (RR 1.40, 95% CI 0.83 to 2.4). The AL rate was 6.11% in LL (23/376) vs. 8.7% in HL (33/379).

The risk of judgment bias was unclear for the different rates of covering stoma. The presence or absence of a stoma was not reported in three studies [33, 43, 44] and was not homogeneous in the other studies.

### Secondary outcomes

#### Overall number of harvested lymph nodes

**Fig. 3:**
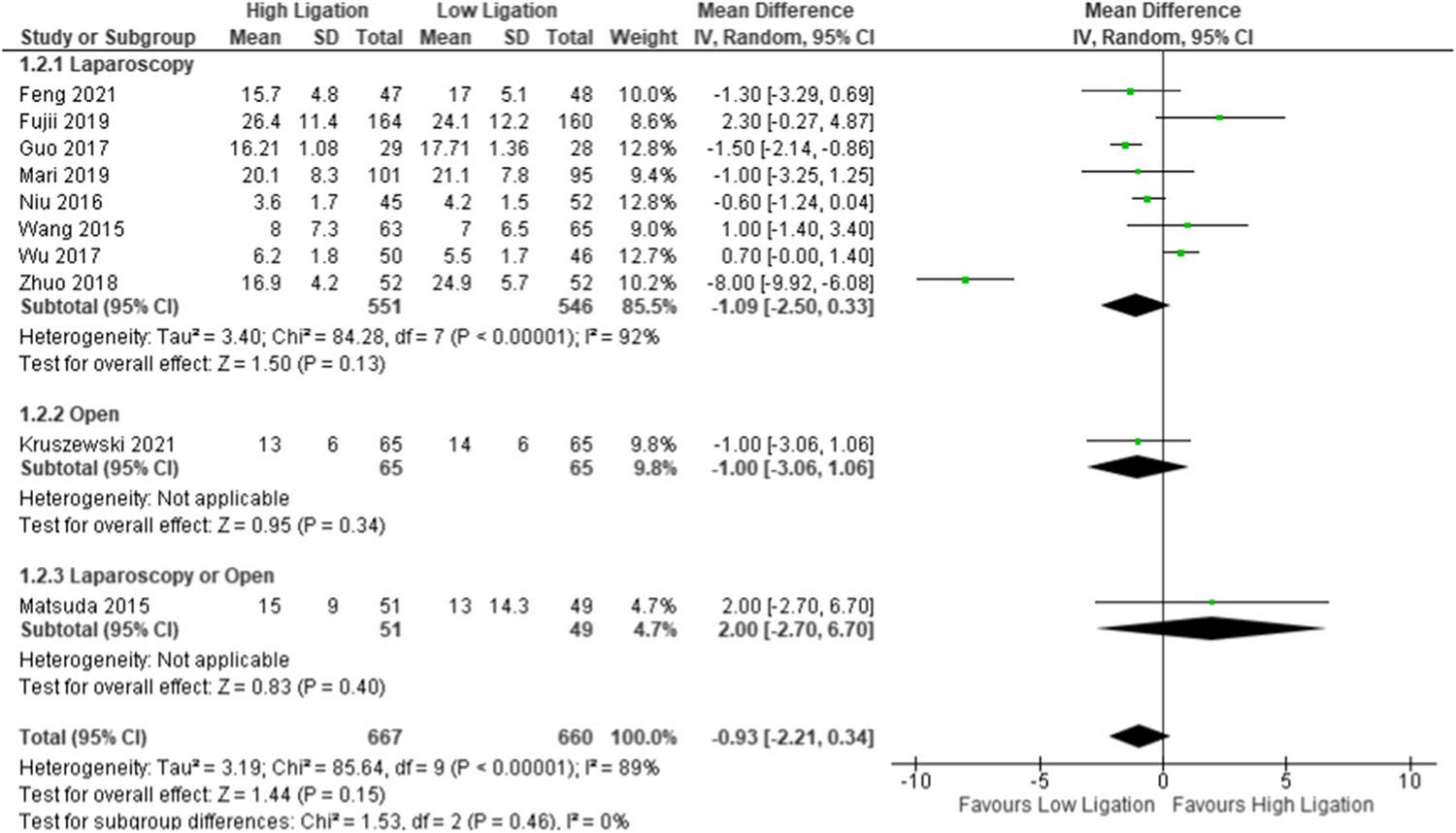
Overall number of harvested lymph nodes

Ten studies reported this outcome (1327 patients: 667 HL vs. 660 LL). The overall number of harvested lymph nodes was higher in the LL group, but the MD was not statistically significant (MD 0.93, 95% CI − 2.21 to 0.34). The heterogeneity was significantly high: Tau^2^ = 3.19; Chi^2^ = 85.84; df = 9 (*P* = 0.00001); *I*^2^ = 89% (Fig. 3). The funnel plot shows an asymmetrical shape, indicating that there was a publication bias [SDC4]; in fact, the results of Zhou et al.’s study [45] were quite different from other studies; for this reason, we conducted a sensitivity analysis to explore the impact of the potential bias associated with the study of Zhou et al. [45]. The new forest plot reported the same result as the previous analysis; in fact, the number of harvested lymph nodes was higher in the LL group, but the result was not statistically significant (MD 0.26, 95% CI − 1.12 to 0.59) [SDC5].

The subgroup analysis of the laparoscopic group reported the same trends favoring LL, but the results were not statistically significant (MD − 1.09, 95% CI − 2.50 to 0.33); furthermore, the sensitivity analysis of the laparoscopic group reported the same result (MD − 0.25, 95% CI − 1.19 to 0.69).

#### Vascular root lymph node harvest

Three studies reported this outcome (participants = 256 patients), and all patients underwent a laparoscopic procedure. The number of lymph nodes was higher when LL was associated with lymphadenectomy of the vascular root than when IMA was ligated at its origin, but the difference was not statistically significant (MD − 0.37, 95% CI − 1.00 to 0.26) [SDC6]. The overall evaluation of the risk of bias was “some concern.” The heterogeneity was significantly high: Tau^2^ =  − 0.26; Chi^2^ = 16.65; df = 2 (*P* = 0.0002); *I*^2^ = 88%.

#### Overall survival at 5 years

**Fig. 4:**
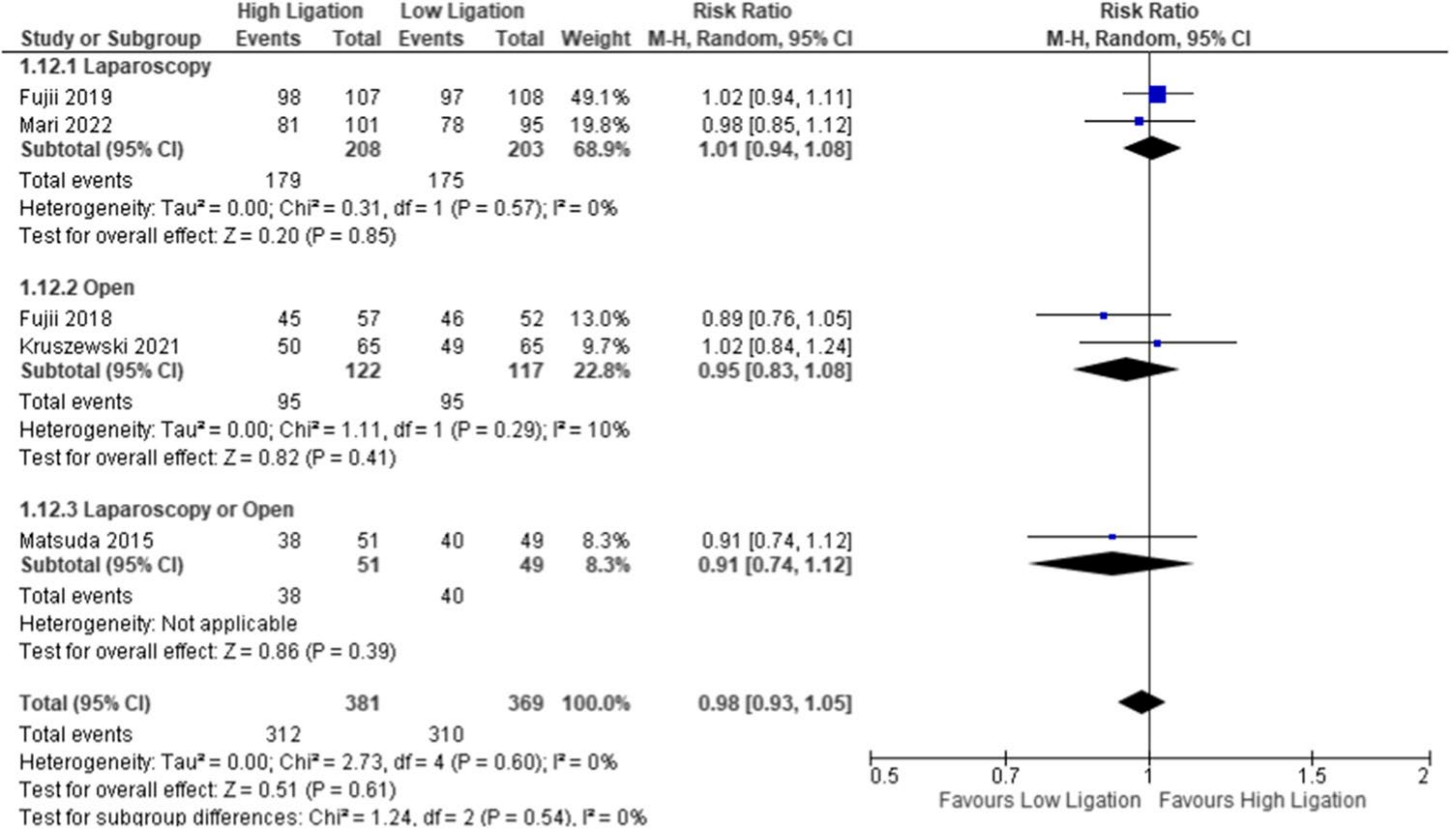
Overall survival at 5 years

Four studies reported this outcome at 5 years (*n* = 750 patients). OS was better in the LL group (84%, 310/369) compared to the HL group (81.89%, 312/381), but the difference was not statistically significant (RR 0.98, 95% CI 0.93 to 1.05) (Fig. 4). The risk of bias was judged as unclear as the authors did not define the modality of oncological follow-up. Heterogeneity was absent (*I*^2^ = 0%). The subgroup analysis of the laparoscopic and the open groups reported the same trends favoring LL. The funnel plot shows a symmetrical shape, indicating that there is no bias [SDC7].

#### Disease-free survival at 5 years

Four studies reported this outcome at 5 years (*n* = 750 patients). The DFS was higher in the LL group (79.4%, 293/369) compared to the HL group (76.64%, 292/381), but the difference was not statistically significant (RR 0.97, 95% CI 0.89 to 1.04) [SDC8]. The risk of bias was judged as unclear as the authors did not define the modality of oncological follow-up. Heterogeneity was absent (*I*^2^ = 0%). The subgroup analysis of the laparoscopic and open groups reported the same trends favoring LL.

#### Conversion from laparoscopic to open surgery

Six studies reported this outcome (764 patients underwent laparoscopic surgery). The conversion from laparoscopic to open surgery rate was lower in the LL group (3.13%, 12/383) (RR 1.45, 95% CI 0.62 to 3.38) than in the HL group (4.72%, 18/381) [SDC9], but the difference was not statistically significant. Heterogeneity was low (*I*^2^ = 18%).

#### Operative time

**Fig. 5:**
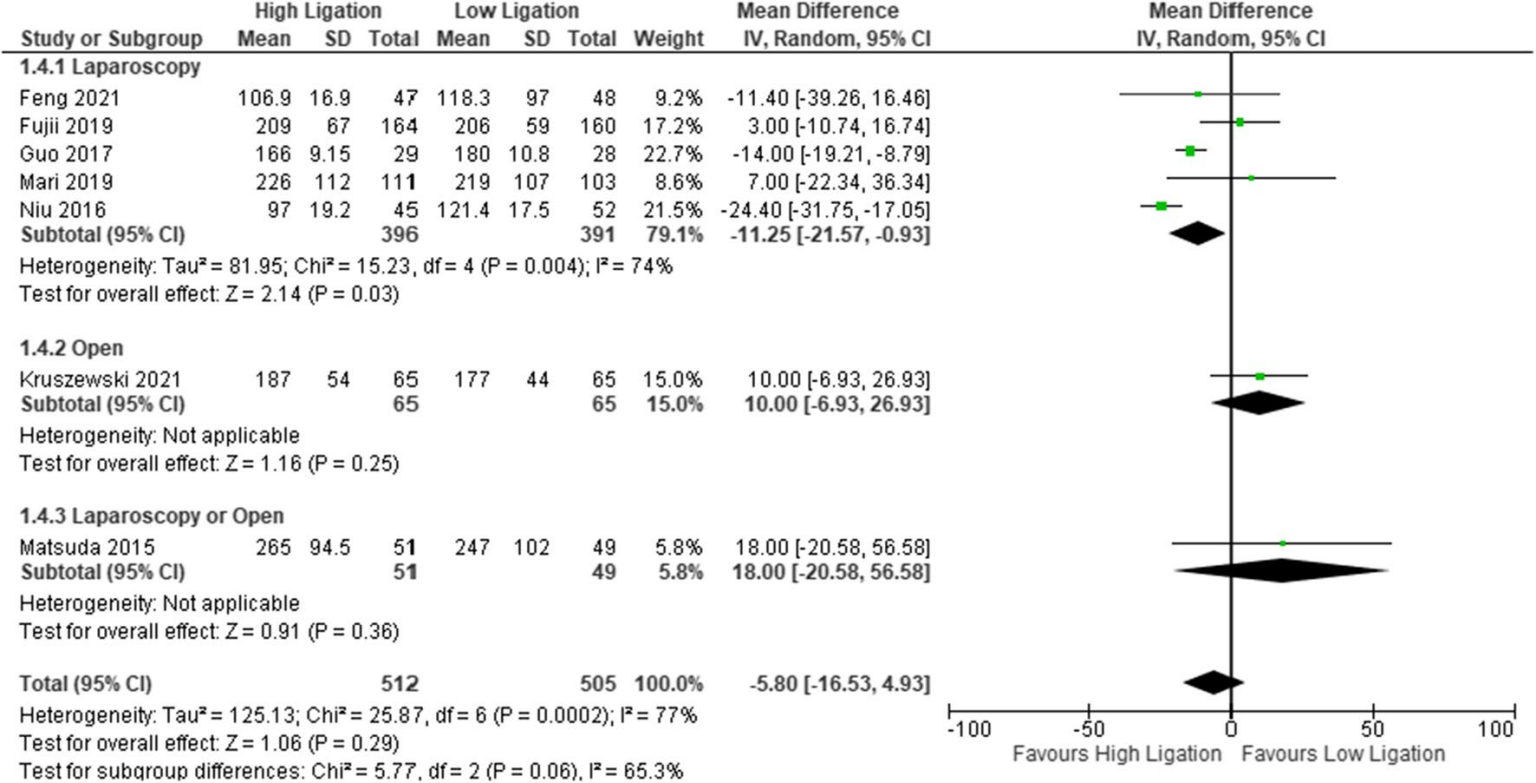
Operative time

Seven studies reported this outcome (*n* = 1017 patients). The authors did not clearly define the parameters for calculating the time span, and for this reason, it was judged an unclear risk of bias. The operative time was significantly lower in the LL group than in the HL group (MD − 5.80, 95% CI − 16.53 to 4.93) (Fig. 5); this trend was the same in the laparoscopic group analysis, but it was reversed in the open group. The heterogeneity was high (*I*^2^ = 77%) [SDC10]. The funnel plot shows a symmetrical shape, indicating that there is no bias.

#### Blood loss

**Fig. 6:**
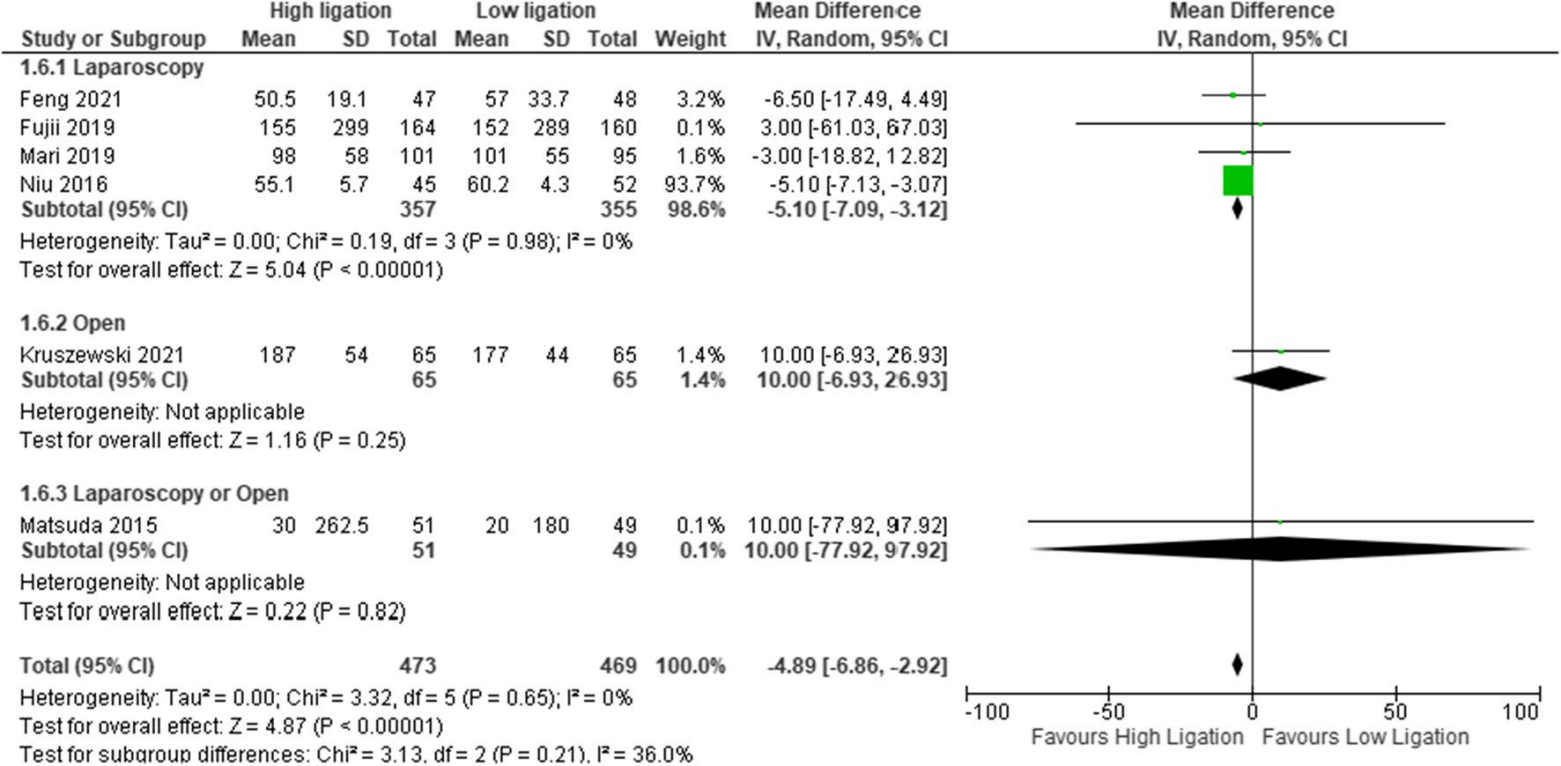
Blood loss

Six studies reported this outcome (*n* = 942 patients). The authors did not report the methodology for the calculation of estimated blood loss, and for this reason, it was judged an unclear risk of bias. The estimated blood loss was higher in the LL group than in the HL group (MD − 4.89, 95% CI − 6.86 to − 2.92) (Fig. 6), but the difference was not statistically significant, and heterogeneity was absent (*I*^2^ = 0%).

#### Urinary retention

Two studies reported this outcome (*n* = 141 patients). The post-operative urinary retention rate was comparable in the two groups (MD 1.09, 95% CI 0.24 to 5.02) [SDC11]. Heterogeneity was absent (*I*^2^ = 0%).

#### Urinary incontinence

Two studies reported this outcome (*n* = 242 patients). This outcome was evaluated using the ICIQ-indicates International Consultation on Incontinence Questionnaire. The results showed a significantly worse result for the LL group if confronted with HL (MD − 0.66, 95% CI 0.92 to 0.40) [SDC12].

#### Sexual dysfunction

Two studies reported this outcome (*n* = 158 patients). The results favored the LL group, although the result was not statistically significant (MD 0.90, 95% CI 0.57 to 1.23) [SDC13]. Heterogeneity was absent (*I*^2^ = 0%). Female sexual function was not assessed in the studies considered.

#### Post-operative length of stay

Two studies reported this outcome (*n* = 419 patients). The length of post-operative stay was lower in the LL group, but the difference was not statistically significant (MD 0.29, 95% CI − 1.01 to 1.59; *I*^2^ = 0%) [SDC 14].

## Discussion

This meta-analysis was not able to show any statistically significant difference in the anastomotic leakage rate, the number of root lymph nodes, the number of lymph nodes retrieved, or overall or disease-free survival in patients with HL or LL.

Anastomotic leakage is one of the most feared complications for patients and surgeons. The pathogenesis of AL is multifactorial and involves tension on the anastomosis, insufficient blood supply, extensive blood loss with the need for transfusion, prolonged operating time, use of inotropes, and impairment of the gut microbiome.

Fashioning a tension-free anastomosis has been traditionally considered a crucial step to reduce the risk of AL. Theoretically, HL should allow a more complete mobilization of the remaining colon and its mesentery, thus preventing anastomotic tension. In fact, the HL of the IMA, possibly associated with the detachment of the LCA from the superior rectal artery, should provide an adequate length of the proximal colon to allow tension-free anastomosis. Conversely, while tension-free anastomosis is fully advisable, avoiding ischemia of the colonic conduit is also necessary to reduce the risk of leakage. When the IMA is ligated at its origin, the blood supply of the descending colon is completely dependent on the marginal artery that arises from the middle colic artery. Inadequate collateral circulation can lead to colonic ischemia in some patients and thus increase the risk of AL. This risk is of greater relevance in elderly patients, where Riolan’s arch may not be completely reliable. The definition of high or low IMA ligation varies somewhat in the literature and is noted elsewhere [9].

According to the Consensus Statement of Definitions for Anorectal Physiology and Rectal Cancer of the American Society of Colon and Rectal Surgeons (ASCRS) (Washington, D.C., May 1, 1999), a low ligation of the IMA is defined as a ligation below the origin of the left colic artery (LCA), while a high ligation of the IMA at the aortic root [47], but leaving a 1.5–2 cm stump to avoid the autonomic nerves of the pre-aortic plexus in the axilla of Bacon.

A tailored approach was suggested by the ASCRS in the practice parameters for the management of rectal cancer. High ligation of the IMA at its origin is not associated with any survival advantage [48], but it is still recommended when clinically enlarged lymph nodes are visible up to the root of the IMA [49].

Factors that may influence the decision to perform a high or low ligation are diverse and involve technical aspects, oncologic outcomes, and functional results, but the choice can be limited by anatomical factors.

In the present analysis, no statistically significant differences were found between the high and low ligation of the IMA with respect to the AL rate. As the HL of the IMA is only one of the possible technical steps to allow a sufficient length of the transposed transverse and proximal descending colon to reach the pelvis, this is perhaps not unexpected. A thorough mobilization of the splenic flexure and the distal transverse colon, along with mobilization of the root of the distal transverse mesocolon and division of any adhesions between the distal transverse and the proximal descending colon, is usually enough to gain an appropriate length of the bowel [50].

Operative time was longer in the HL group than in the LL group. This difference remained unchanged in the analysis of the laparoscopic group, while it was the opposite in the open group, probably due to the technique used. It is difficult to explain this finding. It would be expected that laparoscopic dissection within the sigmoid mesocolon to prepare the bifurcation of the IMA would take longer than preparing the root of the IMA just distal to the aorta, as it is in open surgery. We wonder if in laparoscopic surgery extra time is necessary to access this peri-aortic plane, whereas a higher level can be more easily reached. However, this minor difference did not affect the likelihood of AL.

Similarly, there was less estimated blood loss in the HL group than in the LL group. This could be due to the dissection being conducted in an avascular plane when the IMA is ligated at its origin, while the preparation of the bifurcation of the IMA requires additional dissection within the mesosigmoid and descending mesocolon. However, this does not seem to have an impact on the AL rate.

A possible confounding factor for our analysis is represented by the eventual fashioning of a stoma; in fact, some authors fashioned a diverting stoma for the following reasons: lower rectal cancer [38, 40, 41], pre-operative chemoradiation [41], suspicion of poor anastomosis [39, 42], narrow male pelvis [35], positive air leak test [35], and partial bowel obstruction before surgery [38, 39]. Also, the rate and type of covering stoma were very heterogeneous. The most common covering stoma was a diverting ileostomy, but the reasons why it was packed and the rate of patients in whom it was done are very divergent among the authors, varying from 100% of patients in Mari et al. [40] down to 7.1 for HL and 4.3 for LL in Kruszewski et al. [39]. Moreover, in surgical practice, we need to consider the additional types of anastomotic protection used (transverse colostomy, trans-anastomotic tube) or the fact that the mechanism in use was not indicated in the original publication. This complicates the homogenization of data and the drawing of conclusions on the impact of ostomy packing as a parallel factor independent of the type of IMA ligation, the subject of examination in this study.

Dissection at the root of the IMA and its ligation there can potentially expose the nerves of the aortic plexus and the hypogastric nerves, especially on the left side, to iatrogenic injury at the axilla of Bacon. For this reason, it has been traditionally suggested not to perform any dissection at the very acute angle between the IMA and aorta but to divide the IMA 1.5–2 cm from its origin. One rationale for a more peripheral dissection, and LL of the IMA, is nerve preservation, and the possibility of avoiding urinary and sexual dysfunctions and their subsequent negative impact on quality of life (QoL).

The present analysis reported that the post-operative urinary retention rate was similar in the two groups. However, it must be emphasized that post-operative urinary retention is not commonly due to nerve injury but to paralysis of the bladder sphincter. Urinary incontinence and erectile dysfunction are the typical consequences of nerve injury to the pre-aortic and hypogastric plexuses. Surprisingly, in this analysis, the post-operative urinary incontinence rate was in favor of the HL group, while the post-operative male sexual function was better preserved in the LL group. However, these results come from a study with very small sample size. In actual fact, few studies evaluated the QoL in patients who underwent rectal resection according to the two types of ligations. Moreover, a limitation of this and most of the studies included in our analysis is that data regarding this outcome are available only from male patients, so it is not possible to draw conclusions on functional and sexual outcomes in females.

One of the key performance indicators in surgical oncology is the level of lymph node (LN) dissection. It is still unclear if the nodal dissection should be extended more centrally (N3) instead of limiting it distally to the origin of the LCA (N2).

Surgeons from Japan consider N3 dissection beneficial in terms of OS and DFS if the local spread of the tumor is greater than T2 or if clinically evident nodal metastases are present [41, 51]. However, the USA guidelines recommend a low dissection in most cases of rectal cancer [52], because nodal metastases at the root of the IMA are relatively uncommon. Furthermore, in patients with central nodal metastasis, the 5-year survival rate is quite low (ranging from 0 to 40%) [41], suggesting that resection of those LNs does not improve survival. Positive central nodes should be considered systemic metastases [24, 53, 54].

The overall number of harvested lymph nodes was higher in the LL group, but no significant differences were reported. Once again, it is difficult to explain why a more distal dissection would yield a higher number of lymph nodes. However, the main determinant of survival was not the level of vascular ligation but the quality of the pelvic/mesorectal dissection [49].

One of the reasons for the high heterogeneity of the analysis of the vascular root-harvested lymph nodes is probably associated with the different extensions of lymph node dissection in the two techniques of HL and LL. In fact, Feng et al. [33] described only a lymph node dissection at the root of the IMA. However, Guo et al. [38] and Zhou et al. [45] reported lymphadenectomy at station n. 253 in LL, meaning this allowed additional lymphadenectomy proximal to the level of IMA division.

An important bias is the absence of a description of vascular anatomical variants of the left colic artery (LCA), reported in only a few of the RCTs [39, 40]. The absence of the LCA is the most important variant, and it is reported at 1.2% (pooled prevalence estimate) in a systematic review and meta-analysis performed in 2.040 patients [55]. This rare variant may be associated with a major risk of AL as a result of insufficient vascularization of the proximal colonic conduit and/or a different lymphatic network, and importantly, this anatomical variation can be assessed pre-operatively on detailed staging CT scan imaging.

High-IMA ligation tends to be more standardized as it is less affected by individual anatomical variability and facilitates apical nodal dissection, as mentioned above. However, the origin of the IMA is technically below the Gerota’s fascia, and preparing the very origin at the beginning of the dissection may lead to the wrong dissection plane.

One of the traditionally advocated advantages of LL is a better vascular supply of the colonic conduit through the LCA, while HL would reduce the blood supply to the remaining colon, in particular in elderly patients with an insufficient marginal arcade [38]. For this reason, in patients without evidently enlarged nodes at the IMA root, LL is recommended, while LCA is identified and protected throughout the operation. However, in cases of very low anastomosis, the presence of an intact LCA may not allow sufficient length of the colonic conduit to reach the pelvic floor. In these cases, after undertaking the other standard maneuvers to mobilize the splenic flexure and mesocolon, it might be necessary to also divide the LCA at its origin from the IMA. If this choice is taken later during the operation, namely after the excision of the specimen, it may be difficult to complete the dissection of the IMA root and the ligation of the LCA. Also, for this reason, it may also be advisable to perform a complete lymphadenectomy of the root of the IMA when a LL is performed. The risk of proximal colonic conduit ischemia with HL may be potentially mitigated with the use of indocyanine green fluorescence [56].

In the recent literature, there are already several systematic reviews and meta-analyses published regarding the optimal ligation level of IMA (Table 3) [13, 16, 57–66]. This systematic review and meta-analysis are to summarize the current scientific evidence regarding the impact of the level of inferior mesenteric artery (IMA) ligation on post-operative and oncological outcomes in rectal cancer surgery; the results of this last review differ from previous publications for the higher number of randomized patients enrolled in short‐ and long‐term outcomes. The enrolment of new RCTs and the long-term results published from old RCTs permit to have a growing body of rigorous data to guide the surgical approach to IMA.

**Table 3:** Systematic reviews and meta-analysis published in the last 10 years

Authors and year of publication	Numbers of RCTs included	Numbers of CCTs included	Functional outcomes	Surgical short-term outcomes	Oncologic long-term outcomes
Tryliskyy et al. (2022)	3	0	X	-	-
Kim et al. (2022)	12	0	X	X	X
Kong et al. (2021)	5	0	-	X	X
Bai et al. (2021	5	9	X	X	X
Jonnada et al. (2021)	31		X	X	X
Yin et al. (2021)	4	17	X	X	X
Si et al. (2019)	6	24	X	X	X
Yang et al. (2019)	4	20	X	X	X
Cui et al. (2019)	2	10	X	X	X
Zeng and Su (2018)	4	14	X	X	X
Yang et al. (2018)	0	8	-	X	X
Fan et al. (2018)	1	16	X	X	X

*RCT*, randomized control study; *CCT*, clinical control study

In the analysis of the overall number of harvested lymph nodes, the result of Zhou et al.’s RCT [45] was quite different from other studies. More total lymph nodes [(24.9 ± 5.7) vs. (16.9 ± 4.2), *P* = 0.001] and No. 253 lymph nodes [(2.4 ± 1.1) vs. (1.5 ± 0.8), *P* = 0.001] were harvested in the HL group as compared to the LL group. The higher favorable results in favor of HL can be explained by the very high laparoscopic skill of colorectal surgeons, that received a grant from the Guangzhou Important Special Program of Health Medicine Cooperation and Innovation (Grant number: 201604020005).

Regarding anastomotic leakage, the type and severity of the complication were not reported in three studies (277 patients) [42, 44, 45]. Some of these leaks might have been type A, without any clinical impact.

We recognize that the *I*^2^ statistic is a relative measure and does not measure the scale of the effect size parameter [67]. Moreover, it is not reliable when the number of included studies is small [68]. The heterogeneity statistic *I*^2^ can be biased in small meta-analyses, which was our case. This is why we should rely on the Tau^2^ statistic and prediction intervals [46, 56].

Data were occasionally sparse for several endpoints. This is why we used the Mantel–Haenszel method [69, 70], a fixed-effect method programmed in RevMan.

In conclusion, the optimal level of IMA ligation should be chosen case by case on the basis of several considerations, taking into account expected functional and oncological outcomes and also considering technical and anatomical issues. However, from the oncologic and AL points of view, there is no evidence to support either of the two approaches. Since only a very limited number of studies focused on the functional aspects following rectal resection, there is insufficient evidence to link functional outcomes to the level of IMA ligation.

### Supplementary Information

Below is the link to the electronic supplementary material.

Supplementary file1 (DOCX 31 KB)

Supplementary file2 (DOCX 188 KB)

Supplementary file3 (DOCX 18 KB)

Supplementary file4 (DOCX 17 KB)

Supplementary file5 (DOCX 33 KB)

Supplementary file6 (DOCX 23 KB)

Supplementary file7 (DOCX 17 KB)

Supplementary file8 (DOCX 31 KB)

Supplementary file9 (DOCX 25 KB)

Supplementary file10 (DOCX 17 KB)

Supplementary file11 (DOCX 23 KB)

Supplementary file12 (DOCX 19 KB)

Supplementary file13 (DOCX 19 KB)

Supplementary file14 (DOCX 23 KB)

## Data Availability

All the data used in the article can be obtained directly from Prof. Roberto Cirocchi MD, PhD (roberto.cirocchi@unipg.it) as the corresponding author.
