# Supplementary figures and images for: Inferior mesenteric artery ligation level in rectal cancer surgery: still no answer—a systematic review and meta-analysis
Source: Langenbecks Arch Surg. 2023 Jul 26;408(1):286. doi: 10.1007/s00423-023-03022-z (PMC10371924; doi:10.1007/s00423-023-03022-z)

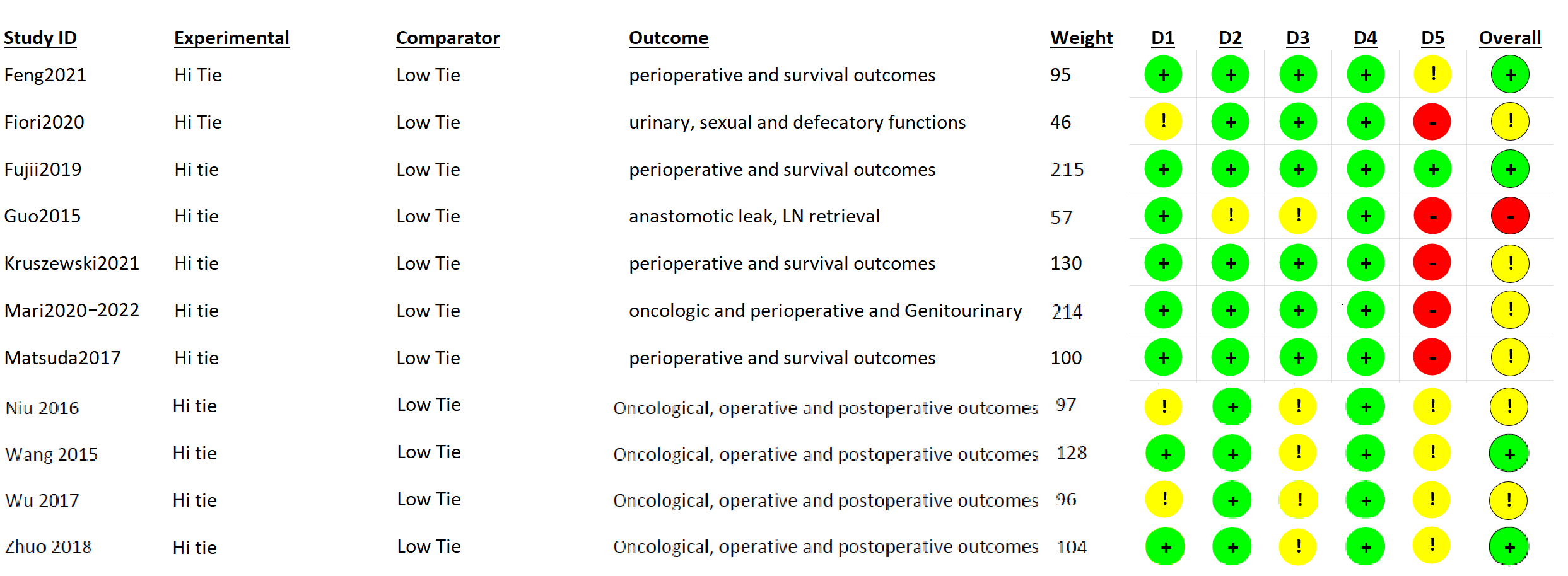


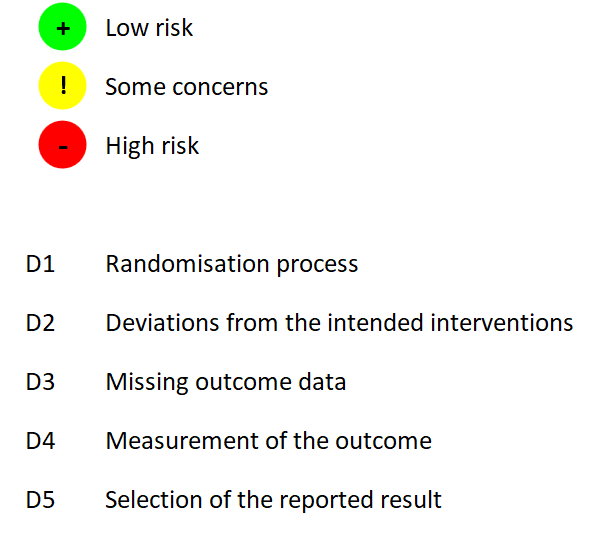

Supplement: Supplementary file 2 — Supplementary file2 (DOCX 188 KB) [file 423_2023_3022_MOESM2_ESM.docx]

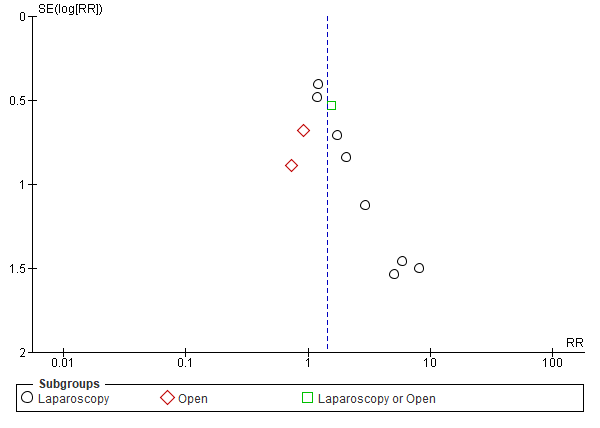

Supplement: Supplementary file 3 — Supplementary file3 (DOCX 18 KB) [file 423_2023_3022_MOESM3_ESM.docx]

**
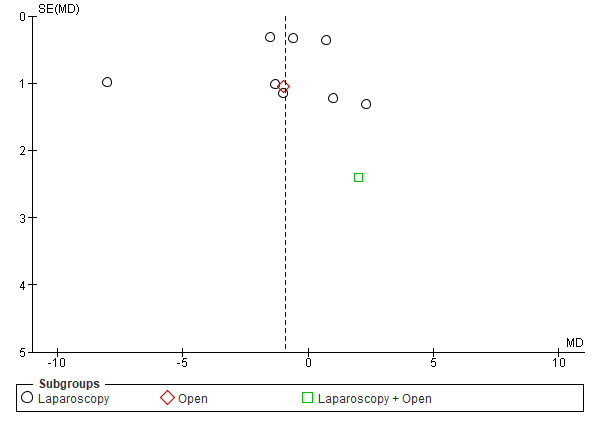
**

Supplement: Supplementary file 4 — Supplementary file4 (DOCX 17 KB) [file 423_2023_3022_MOESM4_ESM.docx]

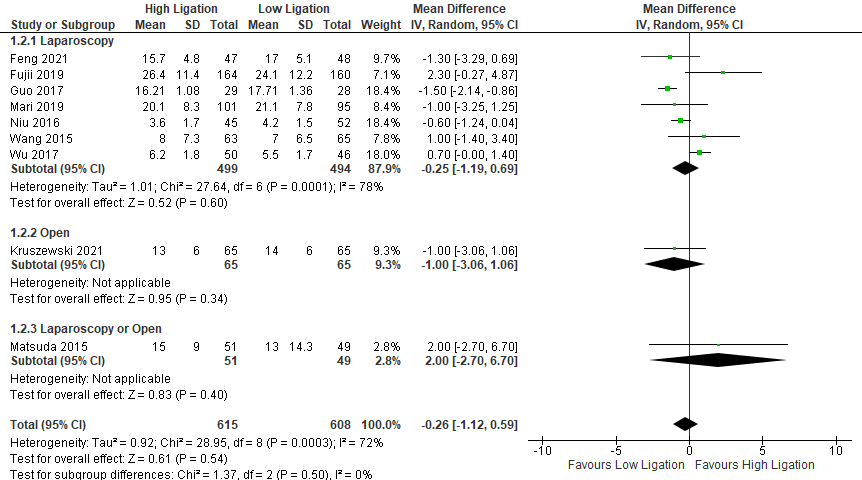

Supplement: Supplementary file 5 — Supplementary file5 (DOCX 33 KB) [file 423_2023_3022_MOESM5_ESM.docx]

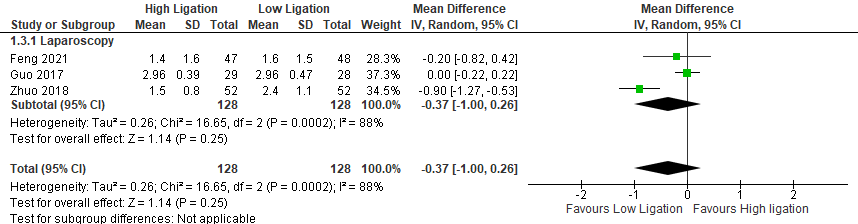

Supplement: Supplementary file 6 — Supplementary file6 (DOCX 23 KB) [file 423_2023_3022_MOESM6_ESM.docx]

**
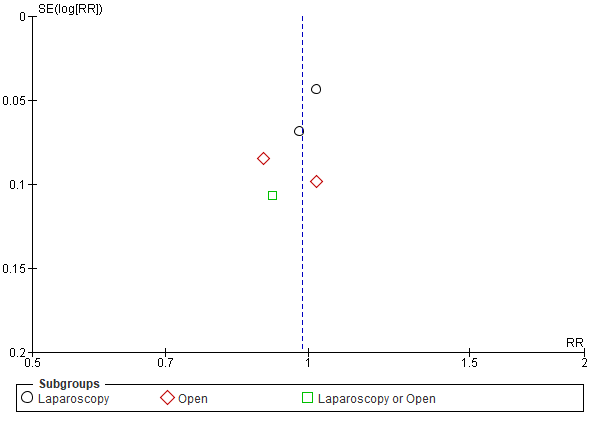
**

Supplement: Supplementary file 7 — Supplementary file7 (DOCX 17 KB) [file 423_2023_3022_MOESM7_ESM.docx]

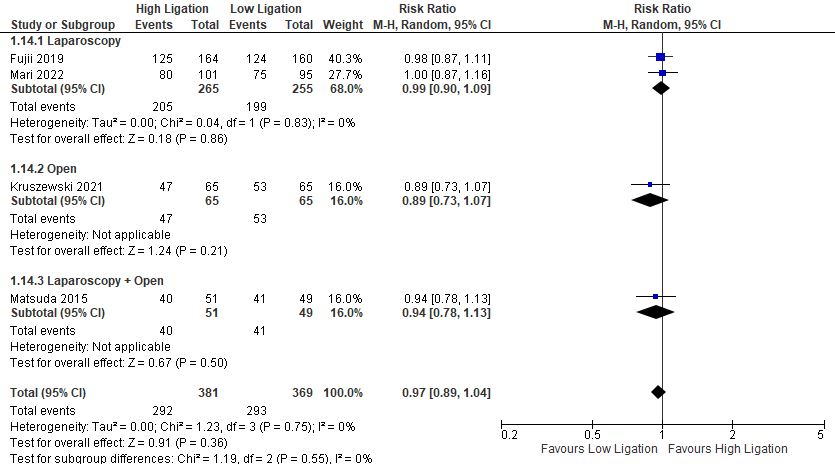

Supplement: Supplementary file 8 — Supplementary file8 (DOCX 31 KB) [file 423_2023_3022_MOESM8_ESM.docx]

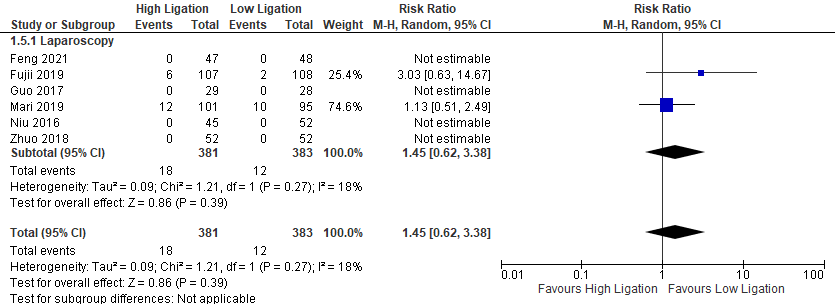

Supplement: Supplementary file 9 — Supplementary file9 (DOCX 25 KB) [file 423_2023_3022_MOESM9_ESM.docx]

**
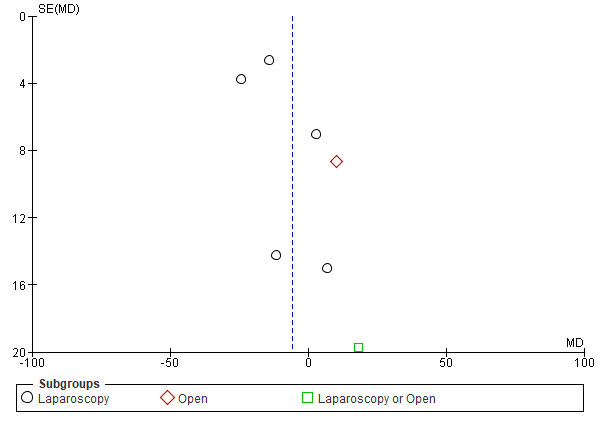
**

Supplement: Supplementary file 10 — Supplementary file10 (DOCX 17 KB) [file 423_2023_3022_MOESM10_ESM.docx]

**
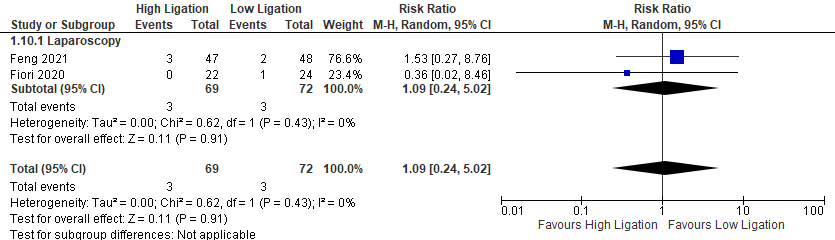
**

Supplement: Supplementary file 11 — Supplementary file11 (DOCX 23 KB) [file 423_2023_3022_MOESM11_ESM.docx]

**
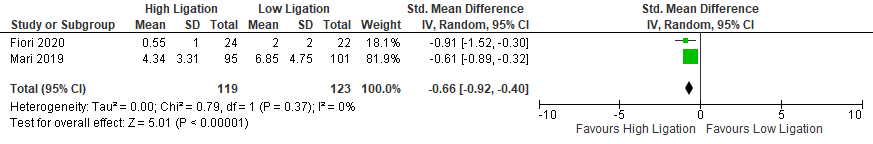
**

Supplement: Supplementary file 12 — Supplementary file12 (DOCX 19 KB) [file 423_2023_3022_MOESM12_ESM.docx]

**
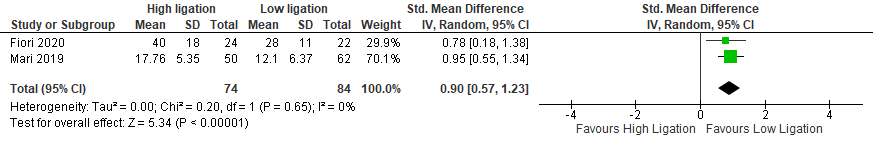
**

Supplement: Supplementary file 13 — Supplementary file13 (DOCX 19 KB) [file 423_2023_3022_MOESM13_ESM.docx]

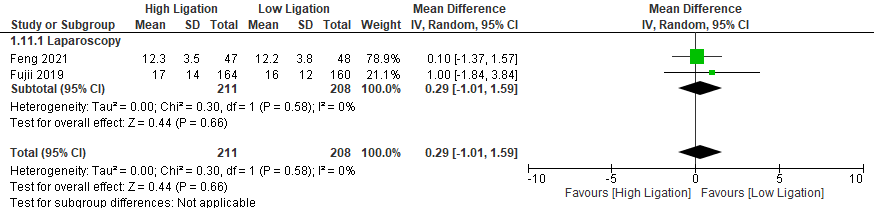

Supplement: Supplementary file 14 — Supplementary file14 (DOCX 23 KB) [file 423_2023_3022_MOESM14_ESM.docx]
